# Interferon Gamma-Based Detection of Latent Tuberculosis Infection in the Border States of Nuevo Leon and Tamaulipas, Mexico

**DOI:** 10.3389/fpubh.2015.00220

**Published:** 2015-09-30

**Authors:** Eyal Oren, Gabriela Alatorre-Izaguirre, Javier Vargas-Villarreal, Maria Guadalupe Moreno-Treviño, Javier Garcialuna-Martinez, Francisco Gonzalez-Salazar

**Affiliations:** ^1^Division of Epidemiology and Biostatistics, University of Arizona, Tucson, AZ, USA; ^2^Health Division, Basic Sciences, University of Monterrey, San Pedro Garza Garcia, Mexico; ^3^Ministry of Health Tamaulipas, Ciudad Victoria, Mexico; ^4^Northeast Biomedical Research, Mexican Social Security Institute, Monterrey, Mexico

**Keywords:** tuberculosis, *Mycobacterium tuberculosis*, tuberculin skin test, interferon gamma, latent tuberculosis infection, Mexico

## Abstract

Nearly one-third of the world’s population is infected with latent tuberculosis (LTBI). Tuberculosis (TB) rates in the border states are higher than national rates in both the US and Mexico, with the border accounting for 30% of total registered TB cases in both countries. However, LTBI rates in the general population in Mexican border states are unknown. In this region, LTBI is diagnosed using the tuberculin skin test (TST). New methods of detection more specific than TST have been developed, although there is currently no gold standard for LTBI detection. Our objective is to demonstrate utility of the Quantiferon TB gold In-Tube (QFT-GIT) test compared with the TST to detect LTBI among border populations. This is an observational, cross-sectional study carried out in border areas of the states of Nuevo Leon and Tamaulipas, Mexico. Participants (*n* = 210) provided a TST and blood sample for the QFT-GIT. Kappa coefficients assessed the agreement between TST and QFT-GIT. Participant characteristics were compared using Fisher exact tests. Thirty-eight percent of participants were diagnosed with LTBI by QFT-GIT. The proportion of LTBI detected using QFT-GIT was almost double [38% (79/210)] that found by TST [19% (39/210)] (*P* < 0.001). Concordance between TST and QFT-GIT was low (kappa = 0.37). We recommend further studies utilizing the QFT-GIT test to detect LTBI among border populations.

## Introduction

Most US–Mexico border states have high rates of unemployment and low per capita income (1). In these states, overcrowding, poor nutrition, and poor access to health services are common (2). Additionally, the US–Mexico border states have rapidly growing populations (3). The US–Mexico border region, extending 37 miles north and south of the border itself, accounts for 30% of total registered tuberculosis (TB) cases in both the US and Mexico (2), and border states from Northern Mexico have the highest rates of TB in the country, with rates between 15 and 39 cases per 100,000 inhabitants (3–6). Those border states with the highest rates, such as in Baja California, Sonora, and Tamaulipas, tend to reflect migration routes from the central and southern Mexican states, as well as Central America, toward the US (7). Additionally, prolonged infectiousness, increased drug resistance, and poor access to health services along the border create barriers to stemming TB disease transmission and providing adequate treatment (8–10). The migratory process itself, poverty, and substandard conditions are likely to be primary factors resulting in increased risk of developing active TB (5, 6, 11).

Infection with *Mycobacterium tuberculosis* bacilli may result in latent tuberculosis infection (LTBI), a carrier state where patients are neither symptomatic nor contagious (12). Latent TB infection is thus frequently not detected or diagnosed. However, when the host immune system fails, infection may progress to active TB disease, at which point symptoms may develop and disease spread can occur (13, 14). The overall lifetime risk of LTBI progression to active TB is estimated at approximately 5–10% (13, 15), with risk increased by underlying immunosuppression, including human immunodeficiency virus (HIV), diabetes, and heavy steroid use, conditions frequently observed along the border (6, 16–19).

Along the Mexico-US border LTBI is diagnosed using the tuberculin skin test (TST), an old diagnostic tool with several limitations in sensitivity and specificity (20, 21). Cross-reactive responses due to BCG vaccination or exposure to environmental mycobacterium can lead to false-positive results, leading to unnecessary follow-up (21). Additionally, high-risk groups such as diabetics, HIV-coinfected, or individuals receiving steroids may not have a detectable TST response due to compromised immune systems (16–19).

In the last few years, in the absence of a gold standard test for LTBI, new methods of detection more sensitive and specific than TST have been developed. These include cell-mediated immunity-based interferon-gamma release assays such as the Quantiferon TB gold In-Tube (QFT-GIT). The QFT-GIT is based on interferon gamma cytokine release in response to TB-specific antigen stimulation of peripheral lymphocytes and has shown equivalent sensitivity and greater specificity than TST in detecting LTBI in several populations (21–23). It is important to note, however, that evaluations of sensitivities provide a suboptimal gold standard, as they utilize clinical situations where a specific immune response is likely to occur, such as among patients with either current active TB or a history of TB disease (21, 23).

Of the studies examining the prevalence of LTBI in the Mexican Border side, the focus has been on high-risk populations. One of these was a study that took place in a migrant agricultural community in San Quintín, Baja California, Mexico. Among 133 participants tested with QFT-GIT, 39.8% had a positive test (24). Another study in Tijuana among 1,020 injecting drug users reported an LTBI prevalence of 67% (25). More recently, a case–control study was carried out among 150 patients from the Mestizo population of Cuidad Juárez, Chihuahua, Mexico (26). Finally, our previously reported study in Nuevo Leon and Tamaulipas showed high LTBI prevalence (~40%) among close contacts to active TB cases (27).

The main objective of this work is to demonstrate utility of the QFT-GIT test to detect LTBI among border populations in Mexico. These outcomes could be used to guide test choice in the future as well as to inform future policy regarding LTBI.

## Materials and Methods

### Study design and inclusion criteria

A cross-sectional study was performed from January through March of 2013 among participants that live in Northern Mexico. Patients from border areas of Nuevo Leon and Tamaulipas were recruited in health clinics belonging to the Nuevo Leon and Tamaulipas Ministries of Health. People living in urban and rural border areas during the above-mentioned period were invited to participate. Patients with active TB disease were referred for follow-up but excluded from the study, although family members of these active TB patients were invited to participate in the study.

Patients diagnosed with cancer, patients diagnosed with HIV, pregnant women, and individuals using steroids for more than 1 month were also excluded. Patients with an incomplete questionnaire or who failed to show for the TST read appointment (48–72 h following the placement) were not included in the data analysis. This protocol was approved by Research and Ethics Committees from the Ministries of Health of Tamaulipas and University of Monterrey (UDEM). All participants, or their parents in case of children, signed an informed consent.

### Questionnaire

Sociodemographic and clinical data were derived through interviewing individuals by trained public health staff using a standardized survey. The survey included medical diagnosis, age, gender, housing conditions, crowding, comorbidities such as diabetes and hypertension, and behavioral risk factors such as smoking, excess alcoholic beverages, or drug use. Responses were all self-reported.

### Procedures

All eligible participants (*n* = 210) were interviewed and then tested for LTBI by TST and QFT-GIT. The QFT-GIT test was performed by trained laboratory personnel who were blinded to the patient’s clinical details. Peripheral blood samples were processed 4–6 h after being obtained from the patient. Initially, the blood was aliquoted into three different tubes: the first did not contain antigens (control), the second tube contained TB antigens, and the third contained phytohemagglutinin (mitogen or positive control) (28). These tubes were incubated for 18–24 h at 37°C. Finally, an ELISA test was performed to determine interferon gamma levels in the plasma of each tube. The results were considered positive, negative, or indeterminate according to the criteria established in the manufacturer’s software. Once the blood was removed to perform the QFT-GIT test, the TST was performed using the Mantoux method, using 0.1 mL (2 tuberculin units) of purified protein derivative RT23 (Statens Serum Institute; Copenhagen, Denmark) in the middle of the anterior face of the forearm, and the outcome was evaluated 48–72 h later by experienced staff. To read the TST, the transverse diameter of the induration was measured and the data registered in millimeters. The TST reaction was scored as positive if the induration diameter was >5 mm. Individuals were considered to have a diagnosis of LTBI if they were asymptomatic without clinical evidence of active TB, but had a positive QFT-GIT and/or TST-positive reaction.

### Statistical analysis

Data were entered into Microsoft Excel 2010. Statistical calculations were made with the statistical package PASW statistics version 18.0. The concordance between the QFT-GIT and TST tests was calculated using statistical kappa (κ). Strengths of agreement were considered “poor” (κ ≤ 0.20), “fair” (0.20 < κ ≤ 0.40), “moderate” (0.40 < κ ≤ 0.60), “good” (0.60 < κ ≤ 0.80), and “very good” (0.80 < κ ≤ 1.00). Descriptive statistics were conducted to describe the sample. Participants were grouped as adult, elderly (60 years of age or more), or pediatric (<18 years of age). Differences in frequencies were evaluated by the Fisher exact test. Statistical significance was defined by *P* ≤ 0.05. Sensitivity and specificity could not be calculated as there is currently no gold standard for LTBI diagnosis.

## Results

### Participants

In this preliminary work in the border states of Nuevo Leon and Tamaulipas, Mexico, we evaluated 210 participants. One hundred and forty-seven of the participants (70%) were in contact with patients with active TB under treatment. The mean age of the participants was 31 years, and almost all (94%) were BCG vaccinated. The proportion of participants with diabetes was 11.9%, while 10% reported suffering from hypertension (Table 1).

**Table 1:** **Patient characteristics (*N* **=** 210)**.

Characteristic	*N*	%
**Age**		
Adults	125	59.5
Elderly	19	9.1
Pediatric	66	31.4
**Gender**		
Male	77	30.6
Female	133	63.4
**BCG History**		
Yes	198	94.3
No	12	5.7
**Diabetes**		
Yes	25	11.9
No	185	88.1
**Hypertension**		
Yes	21	10
No	189	90

*Elderly, 60 years of age or more; pediatric, <18 years of age*.

### LTBI diagnosis

All (210) the participants were QFT-GIT tested, with 207 (99%) completing the TST evaluation. The proportion of LTBI detected using QFT-GIT was close to double [38% (79/210)] that found by TST [19% (39/207)] (*P* < 0.001) (Table 2). The proportion of participants with LTBI detected by QFT-GIT among close contacts was 49.7% (73/147) compared to 21% by TST (31/147). When a positive result on either test was considered evidence of LTBI, 40.9% (86/210) of participants were found to have LTBI. Individuals not considered contacts were only positive by QFT-GIT 9.5% of the time (6/63) (*P* < 0.001) and by TST 12.6% of the time (8/63) (*P* < 0.01).

**Table 2:** **Comparative outcomes from QFT-GIT and TST**.

Distribution of patients	QFT-GIT		*P*-value	TST			*P*-value
	Negative	Positive		Negative	ND	Positive	
	*n* = 131	*n* = 79		*n* = 168	*n* = 3	*n* = 39	
**Age**							
A	81 (64.8%)	44 (35.2%)	0.67	104 (83.2%)	3 (2.4%)	18 (14.4%)	0.05
E	11 (57.9%)	8 (42.1%)		17 (89.5%)	0 (0%)	2 (10.5%)	
P	39 (59.1%)	27 (40.9%)		47 (71.2%)	0 (0%)	19 (28.8%)	
**Gender**							
M	49 (63.6%)	28 (36.4%)	0.44	63 (81.8%)	0 (0%)	14 (18.2%)	0.24
F	82 (61.7%)	51 (38.3%)		105 (78.9%)	3 (2.3%)	25 (18.8%)	
**Contact**							
Yes	74 (50.3%)	73 (49.7%)	0.001*	116 (78.9%)	0 (0%)	31 (21.1%)	0.01*
No	57 (90.5%)	6 (9.5%)		52 (82.5%)	3 (5.8%)	8 (12.7%)	
**BCG**							
Yes	123 (62.1%)	75 (37.9%)	0.50	157 (79.3%)	3 (1.5%)	38 (19.2%)	0.48
No	8 (66.7%)	4 (33.3%)		11 (91.7%)	0 (0%)	1 (8.3%)	
**Diabetes**							
Yes	11 (44%)	14 (56%)	0.03*	150 (81.1%)	3 (1.7%)	32 (17.2%)	0.33
No	120 (64.9%)	65 (35.1%)		18 (72%)	0 (0%)	7 (28%)	
**Hypertension**							
Yes	13 (61.9%)	8 (38.1%)	0.56	152 (80.4%)	3 (1.6%)	34 (18%)	0.60
No	118 (62.4%)	71 (37.6%)		16 (76.2%)	0 (0%)	5 (23.8%)	

*A, adult; E, elderly, 60 years of age or more; P, pediatric, <18 years of age; M, male; F, female; ND, not done*.

*Statistically significant, P < 0.05.

No significant differences were observed across gender. The proportion of participants with positive TST was inversely associated with increasing age (*P* = 0.05). While the proportion of participants testing positive by QFT-GIT was higher among the elderly and children (Table 2), these differences were not statistically significant. The proportion of participants with positive QFT-GIT was significantly higher in people with diabetes (56%) compared to that of non-diabetics (35%) (*P* = 0.03). These differences could not be detected with TST. No differences were found in the proportion of positive QFT-GIT and TST tests among participants with or without hypertension.

### Test concordance

The assays had a concordance of just *k* = 0.37 (*P* < 0.01) with each other among contacts (Table 3), approximately the same as when all asymptomatic people reviewed in the study were included (0.39) (Table 4). When close contacts of pulmonary active TB cases were tested with QFT-GIT and TST, some individuals with positive QFT-GIT had negative TST (45/73) and some with negative QFT-GIT had positive TST results (3/74). In this scenario, only 38.3% (28/73) of individuals with LTBI detected by QFT-GIT had a positive TST test; however, when QFT-GIT was negative (71/74), 95.9% of the individuals were TST negative as well. If the QFT-GIT results are correct, the proportion of false-negative TST tests was 61.6% (45/73) and false positive was 4.1% (3/74) (Table 3).

**Table 3:** **Concordance between TST and QFT-GIT among contacts only**.

Test	QFT-GIT**+**	QFT-GIT**−**	Total
TST+	28	3	31
TST−	45	71	116
Total	73	74	147

**k* = 0.37*.

**Table 4:** **Concordance between TST and QFT-GIT among all participants**.

Test	QFT-GIT**+**	QFT-GIT**−**	Total
TST+	32	7	39
TST−	47	121	168
Total	79	128	207^a^

*k = 0.39*.

*^a^Three TSTs were not read*.

## Discussion

This study showed a high prevalence of LTBI among border populations. An even higher proportion of participants were found to have LTBI when limited to those with close contact to active TB patients. Higher positivity rates were observed by the QFT-GIT test, with many of these individuals displaying a negative TST. Test concordance between the two tests was fairly low.

Others have also shown a prevalence of close to 40% among migrant residents on the US side of the border, in Baja California (24). Given the elevated LTBI prevalence observed, there are likely many individuals who have active TB who are not being diagnosed or treated. Particularly, troubling is the high prevalence of LTBI among high-risk groups for progression to active TB, such as close contacts and individuals with comorbidities such as diabetes. In Mexico, most patients are not offered testing or treatment for LTBI, with the exception of diabetic patients in close contact with active TB patients, who are currently offered preventive treatment regardless of their LTBI status (29). Moreover, Mexican-born TB patients on the US border have been previously reported to have greater disease severity, with more frequent sputum smear-positive and cavitary disease, possibly due to more limited access or delay in seeking care (30). Delayed diagnoses, combined with the lack of LTBI detection, only serve to continue a cycle of disease transmission within and across households.

Given these high prevalence rates, it is essential that screening and appropriate preventive therapy be provided for those at high risk of progression, and perhaps more broadly for workers and their families. However, given the early risk of isoniazid (INH) toxicity, persons diagnosed with LTBI will need to be counseled and instructed to seek medical attention if they develop TB symptoms having declined INH treatment (31). Targeted testing of specific populations at high risk for progression to active disease, such as priority groups that are medically underserved or immune-compromised, may be helpful in curbing the progression to active TB disease (30). However, as has been previously noted, how to best target populations in a systematic manner is still unclear (32, 33).

Quantiferon TB gold In-Tube has previously been shown to have higher specificity compared with the TST when compared to patients with a positive culture, as well as a higher predictive ability that a test-negative individual will not develop disease or that a test-positive individual will progress (22). However, in this study, the proportion of individuals who tested positive by QFT-GIT was greater than those testing positive by TST, indicating that QFT-GIT may be more likely to correctly identify individuals as infected with TB compared to the TST. Given these results, it might be important to consider QFT-GIT among high-risk groups who may not have a detectable TST response, such as diabetics, HIV-coinfected, or individuals receiving steroids or immunotherapy (34). As an example, in populations (such as in Monterrey, Nuevo Leon, Mexico) where the incidence of TB is 20 per 100,000 habitants, this would mean that if each patient had a mean of five contacts, approximately 2,000 contacts would be located in a given year. Given the data from our study, we would then expect 1,200 false-negative test results with the TST. In essence, this means that many individuals who actually have LTBI are left untreated and may subsequently progress to active TB disease. This is particularly true in a population with many comorbidities that decrease immune function (16–19).

As per the authors’ prior results from the Mexico side of the border, we found the TST and QFT-GIT to show a low level of concordance (27), although this was more often due to QFT-GIT+/TST− combinations, rather than the converse, as had previously been reported (27).

Since no systematic procedures have been in place to screen individuals for latent TB infection, we recommend a study to examine the feasibility of providing infected individuals with testing, follow-up screening, and treatment services. The main objective of the proposed study would be to demonstrate the potential utility of the QFT-GIT test to detect LTBI as well as the feasibility of implementation among border populations. Since LTBI is not routinely diagnosed, testing on the border will potentially allow for diagnosis and follow-up prophylaxis. An earlier version of the QFT-GIT has previously been shown to be cost-effective for targeted screening in Mexico (35). Additionally, assessing the LTBI status along the border will both provide an assessment of whether QFT-GIT should replace the TST in routine practice and identify predictive risk factors for LTBI in these populations. These results could be of particular significance because of both the costs and adverse effects of LTBI treatment that might occur among false positives as well as the possible detection using QFT-GIT of immunocompromised individuals (36). We are continuing this work in Mexico and have begun following up on this work in the Yuma/San Luis region in the state of Arizona in the United States.

Limitations of our study include the cross-sectional nature of the results, self-selection of participants into the study, as well as the ability to generalize these results to other parts of the border – the rural farmworkers sampled are more economically stable and far less mobile than the migrant farmworkers observed in many parts of the US and may not be representative of other farmworker groups. Another important limitation is the lack of culture confirmation of TB disease in Mexico. Close contacts were thus selected based on both a self-report and a sputum smear (Ziehl–Nielsen) diagnosis among the active index case, which is not considered confirmatory. Nevertheless, given the high prevalence of LTBI among the close contacts, it is likely that many of the index cases were at some point infectious.

An association has previously been found between travel to the US and the presence of LTBI (8). Initiatives to increase TB awareness and testing and treatment of latent TB infection and disease are thus critical to TB elimination efforts along the border region, as populations repeatedly move and work on both sides of the border. Binational public health action to prevent individuals with LTBI from progressing to TB through proper screening and treatment is essential for TB control and prevention, just as has been advocated for active TB (20). As for active TB, we believe that partnerships should be facilitated between healthcare providers for LTBI across both sides of the US–Mexico border, considering the border region to be one single unit, with documentation of effective strategies for cross-border notification of movement and treatment coordination (7).

Additional next steps based on this work could include providing populations on the border with information around TB and LTBI, including training and informational materials. Increasing service utilization is a key challenge. Poor communication, government mistrust, and misunderstanding of the health system are some of the barriers to effective health service delivery. Explaining the flow of the clinic and health system process for those with TB exposure may provide a significant first step. Such education could be integrated with existing programs and community health center initiatives for individuals living on the border.

## Conflict of Interest Statement

The authors declare that the research was conducted in the absence of any commercial or financial relationships that could be construed as a potential conflict of interest. The Associate Editor Scott C. Carvajal declares that, despite being affiliated with the same institution as author Eyal Oren, the review process was handled objectively and no conflict of interest exists.
